# CCR4-NOT complex in stress resistance and longevity in *C. elegans*

**DOI:** 10.18632/aging.205918

**Published:** 2024-05-16

**Authors:** Cheng-Wei Wu, Hadi Tabarraei

**Affiliations:** 1 Department of Veterinary Biomedical Sciences, Western College of Veterinary Medicine, University of Saskatchewan, Saskatoon, Canada

**Keywords:** aging, oxidative stress, *C. elegans*, CCR4-NOT

The ability to mount an adaptive response to environmental stress is crucial in organismal survival and overall fitness. In the context of aging, many genes that mediate resistance to stressors are also important in longevity, and aging has been shown to cause a decline in stress resistance [1]. In screening for genes that are required for the transcriptional response to heavy metal and oxidative stress in *C. elegans*, we recently found that depletion of subunits within the evolutionarily conserved CCR4-NOT protein complex compromises stress resistance and decreases lifespan [2].

The CCR4-NOT (Carbon Catabolite Repression 4 – Negative On TATA-less) is a multi-protein complex tasked with regulating RNA metabolism across multiple steps including mRNA decay, transcription initiation and elongation, mRNA quality control and export, and mRNA translatability (reviewed in [3]). Studies in yeast have shown that CCR4-NOT is required for transcriptional elongation of stress responsive genes and that loss of function mutants of this protein complex have increased sensitivity to replication stress caused by DNA damaging agents [4, 5]. An expansive role for the CCR4-NOT complex in stress-induced transcriptional programming was demonstrated in *C. elegans* via whole-transcriptome sequencing analysis [2]. RNAi knockdown of *ccf-1* (CCR Associated Factor) encoding the deadenylase subunit of CCR4-NOT caused global down-regulation of cadmium and acrylamide-responsive genes that highly enrich to xenobiotic activity including those functioning in cytochrome P450 and glutathione metabolism. Consistent with the requirement for CCR4-NOT in transcriptional programming, RNAi knockdown of *ccf-1* significantly decreases *C. elegans* survival to cadmium and acrylamide exposure. In a separate *C. elegans* study, Daskalaki et al. found that NTL-2 (Negative on TATA-less-Like) encoding a separate subunit of the CCR4-NOT complex directly associates with the mitochondria, and knockdown of *ntl-2* decreases survival to heat stress and mitochondria inhibitors paraquat and carbonyl cyanide m-chlorophenyl hydrazine [6].

**Figure 1:**
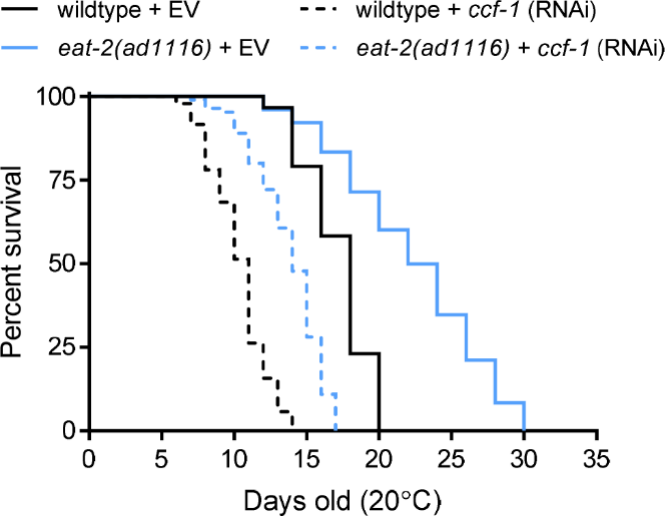
**Effects of *ccf-1* knockdown on the lifespan of N2 wildtype and *eat-2(ad1116)* mutant.** N = 84-121 worms scored per condition. EV = empty vector.

Perhaps the most interesting result from both studies is that the knockdown of *ccf-1* and *ntl-2* drastically decreases *C. elegans* lifespan, suggesting that the CCR4-NOT complex is required for normal aging. Additional analysis also shows that *ccf-1* is required for the lifespan extension of several evolutionarily conserved longevity pathways including the *daf-2(e1370)* (insulin receptor) mutant, *hsf-1(FL)* (heat shock factor) overexpression, *skn-1(k1023)* (Nrf-2) gain of function; meanwhile, *ntl-2* was shown to be required for the longevity of the *age-1(hx546)* (PI3K) mutant and mitochondrial mutants *mev-1(kn1)* (succinate dehydrogenase) and *nuo-6(qm200)* (NADH: ubiquinone oxidoreductase mutant) [2, 6–8]. Furthermore, we have determined that the knockdown of *ccf-1* also attenuates the longevity of the *eat-2(ad1116)* mutant, which is a genetic mimetic of dietary restriction (Figure 1). How does inhibiting the CCR4-NOT complex compromise lifespan? Mechanistically, *ccf-1* is required for the normal expression of a large class of xenobiotic detoxification genes, many of which may be required for normal lifespan. For example, knockdown of *ccf-1* decreases expression of the glutathione s-transferase genes *gst-5* and *gst-10* under stress, both of which have been shown to reduce lifespan when knocked down in *C. elegans* [9]. Furthermore, NTL-2 was shown recently to serve as a storage body for mitochondrial-targeted protein mRNAs, which when knocked down disrupts mitochondrial homeostasis and causes an aberrant increase in global protein synthesis, both of which can be detrimental to longevity [6]. As such, it appears that the CCR4-NOT complex can influence longevity in a multitude of manners, akin to its wide range of roles in maintaining RNA metabolism homeostasis.

Together, while the CCR4-NOT complex has been extensively studied for the past 3 decades, new studies in the model organism *C. elegans* have revealed an important new role for this protein complex in regulating normal aging as well as a requirement for many well-characterized and evolutionarily conserved pro-longevity pathways including reduced insulin signaling, mitochondrial suppression, enhanced stress response, and dietary restriction.
